# Using Functionalized Micron-Sized Glass Fibres for the Synergistic Effect of Glass Ionomer on Luting Material

**DOI:** 10.3390/jfb14110550

**Published:** 2023-11-16

**Authors:** Hanan Alsunbul, Aftab Ahmed Khan, Yasser M. Alqahtani, Saeed Awod bin Hassan, Waleed Asiri, Selma Saadaldin, Rasha Alharthi, Alhanoof Aldegheishem

**Affiliations:** 1 Restorative Dentistry Department, College of Dentistry, King Saud University, Riyadh 11451, Saudi Arabia; halumbol@ksu.edu.sa; 2 Dental Health Department, College of Applied Medical Sciences, King Saud University, Riyadh 11451, Saudi Arabia; 3 Restorative Dentistry Department, Ministry of Health, Abha 61321, Saudi Arabia; yasmhh333@gmail.com; 4 Restorative Dental Sciences Department, College of Dentistry, King Khalid University, Abha 61321, Saudi Arabia; samhasan@kku.edu.sa; 5 Restorative Dentistry Department, College of Dentistry, Najran University, Najran 66454, Saudi Arabia; wsasiri@nu.edu.sa; 6 Prosthodontics Division, Schulich School of Medicine and Dentistry, Western University, London, ON N6A 5B9, Canada; ssaadal@uwo.ca; 7 Clinical Dental Science Department, College of Dentistry, Princess Nourah bint Abdulrahman University, Riyadh 11671, Saudi Arabia; rsalharthi@pnu.edu.sa (R.A.); asaldegheishem@pnu.edu.sa (A.A.)

**Keywords:** glass fibre, glass ionomer luting, functionalization, synergistic effect

## Abstract

This laboratory experiment was conducted with the objective of augmenting the mechanical properties of glass ionomer cement (GIC) via altering the composition of GIC luting powder through the introduction of micron-sized silanized glass fibres (GFs). Experimental GICs were prepared through the addition of two concentrations of GFs (0.5% and 1.0% by weight) to the powder of commercially available GIC luting materials. The effect of GF in set GIC was internally evaluated using micro-CT while the mechanical attributes such as nano hardness (nH), elastic modulus (EM), compressive strength (CS), and diametral tensile strength (DTS) were gauged. Additionally, the physical properties such as water solubility and sorption, contact angle (CA), and film thickness were evaluated. Reinforced Ketac Cem Radiopaque (KCR) GIC with 0.5 wt.% GF achieved improved nH, EM, CS, and DTS without affecting the film thickness, CA or internal porosity of the set GIC cement. In contrast, both GF-GIC formulations of Medicem (MC) GIC showed the detrimental effect of the GF incorporation. Reinforcing KCR GIC with 0.5 wt.% silanized GFs could improve the physical and mechanical attributes of luting material. Silanized GF, with optimal concentration within the GIC powder, can be used as a functional additive in KCR GIC with promising results.

## 1. Introduction

Achieving successful indirect restorations profoundly relies on the precise cementation of indirect restorations to teeth using appropriate luting agents. These types of restoration encompass metal, metal—ceramic, ceramic, veneers for anterior teeth, orthodontic appliances, post and core. The principal role of a luting agent is to fill the gap present at the interface between the restoration and the tooth. Additionally, it serves to mechanically secure the restoration, preventing its displacement during the process of mastication [1].

The dimensional stability of luting agents is a crucial characteristic that significantly impacts the clinical life of dental restorations [2]. The polymerization shrinkage that occurs after the application of resin-based luting materials can result in marginal gaps and leakage, potentially leading to secondary caries and ultimately compromising the integrity of the restoration [3]. Polymerization shrinkage occurs due to changes in molecular bonding, transitioning from van der Waals forces to covalent single bonds, as well as alterations in atomic distances [4]. This reaction also generates heat, especially in the case of light-activated products, due to the additional heat produced by the curing device [5].

While resin-based glass ionomer cement (GIC) presents a viable alternative for luting agents, it is noteworthy that the inclusion of HEMA and the subsequent polymerization process, initiated either via light or chemical means, gives rise to polymerization-induced shrinkage and a consequential elevation in temperature during the setting phase [6]. Due to polymerization shrinkage, the dimensional stability of resin-based GIC is affected and the longevity of the restoration is compromised [7].

GIC, despite having poor mechanical properties [8], has one prominent advantage: fluoride is released over a long period [9,10]. Furthermore, it has been demonstrated that GICs can be recharged with fluoride ions in laboratory settings [11]. In addition, biocompatibility with the pulp and chemical bonding with the tooth structure make GICs popular as luting agents. This is because fluoride has the capacity to inhibit demineralization and facilitate the remineralization of dental hard tissues [12].

In recent years, there has been a notable trend in improving the attributes of GIC materials via the introduction of fillers/fibres such as hydroxyapatite, bioactive glass, biopolymers, nano clay, and discontinuous glass fibres (GFs) [3,13,14] to fortify strength and elevate the elasticity modulus [1]. The use of discontinuous GF reinforcement, i.e., reinforcing GFs with diameters from a few micrometres to twenty micrometres with a high aspect ratio in many fields of dentistry is already established [14]. Due to exceptional biocompatibility associated with many advantages such as reinforcing agents in denture bases, endodontic posts, restorative materials, orthodontic appliances, and periodontal splints, it is the material of choice [15]. However, GF investigation in the context of GIC luting cements remains somewhat limited. Thus, further exploration is necessary to formulate a material with improved mechanical characteristics. Many aspects of fibre composites’ properties are closely tied to factors such as fibre-matrix adhesion and microstructural parameters, including fibre diameter, length, orientation, and loading. [16]. Therefore, this study evaluated the effect of GFs with different loading fractions on select mechanical properties of GIC. To the authors’ knowledge, there is a paucity of research in this specific research area. Thus, the hypothesis stated that the incorporation of randomly distributed GFs could reinforce a GIC luting material.

## 2. Materials and Methods

### 2.1. Preparation of Experimental Glass-Fibre Reinforced GICs

Short electrical grade-GFs having a length scale of 150 μm, Ø 16 μm, and an aspect ratio of 11:1 were used. The GFs were already silanized with 3-[Trimethoxysilyl] propyl methacrylate, MPS and were procured from Sigma-Aldrich (St. Louis city, MO, USA). Experimental GICs were prepared via the addition of short GFs to the powder composition of two commercially available type I GICs, namely Medicem (MC; Promedica Dental Material GmbH, Neumuenste, Germany) and Ketac Cem Radiopaque (KCR; 3M ESPE, Seefeld, Germany) with two different weight ratios (0.5 and 1.0 wt.%). The mixing was performed via an amalgamator (Promix^TM^; Dentsply Caulk, York, PA, USA) for 20 s to achieve a homogenous powder mixture. The control groups for both GICs were prepared without GF (0 wt.%). The powdered composition for both the control and experimental GICs was blended with the liquid component using the prescribed powder-to-liquid ratio of 1:1.

**Figure 1:**
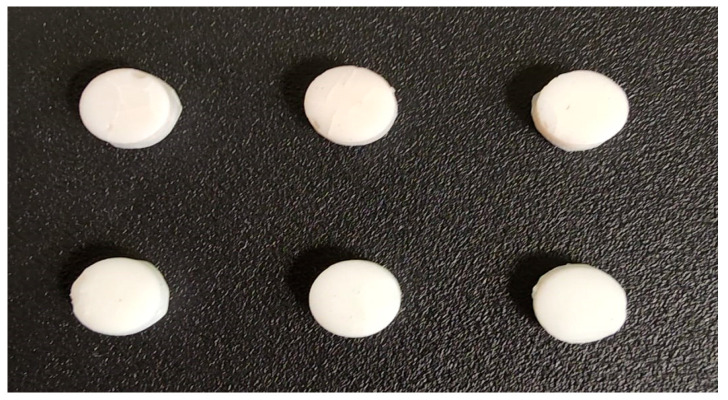
Circular-shaped study specimens with 0%, 0.5% and 1.0% wt.% GF incorporated in GIC (from left to right). The upper row denotes specimens prepared with KCR, while the lower row links to specimens prepared with MC.

Circular-shaped specimens with a 6 mm diameter and 3 mm height (as illustrated in Figure 1) were fabricated. The specimen fabrication process involved the thorough mixing of powder and liquid components until it achieved a paste-like consistency, which was then poured into silicon moulds. After 30 min, the specimens were carefully released from the moulds and placed in appropriately labelled containers. These specimens were subsequently stored in an incubator set at 37 °C for a duration of 7 d before undergoing a series of tests to assess their physical and mechanical properties. Importantly, all specimen fabrications were executed by a single trained operator under a controlled room temperature of 22 °C.

### 2.2. Visual Evaluation

A representative specimen from each study group was evaluated for surface texture and finish using a light stereomicroscope (Nikon SM2-10, Tokyo, Japan) at a magnification of ×20.

### 2.3. Micro-Computed Tomography (Micro-CT) Test

A single specimen from each study group was selected to assess the three-dimensional internal structure and flaws of the study specimens. A micro-computed tomography system (Skyscan 1172, Bruker, Aartselaar, Belgium) was used to evaluate the porosity percentage in each specimen. The scanning parameters were set at a voltage of 100 kV, current of 50 μA, voxel size of 14.2 μm and 0.5 rotation step for 160° capturing 902 projections. These configurations were selected to detect the possible pores in the specimens. A pore was referred to as ‘closed’ when it was enclosed within the material and was not connected to the external environment. While void spaces that were connected to the external environment or to neighbouring pores were termed as open pores. The N-Recon^®^ software ver. 1.6.1.3 (Bruker Skyscan, Kontich, Belgium) was used for 3D image reconstruction. For visual inspection, the Dataviewer^®^ program ver. 1.5.6.2 (Bruker Skyscan, Kontich, Belgium) was used. Finally, the CTAn© ver. 1.20.8.0 (Bruker Skyscan, Kontich, Belgium) software was used to randomly inspect the area of interest measuring 3 mm × 1.5 mm to calculate the porosity values. The porosity was determined in relation to the entire dataset, encompassing the air surrounding the specimen, thereby resulting in an estimation of porosity and pore volume. An appropriate threshold was established for the selection of specific structures based on their respective grey values. Subsequently, the 3D analysis option was employed to calculate the pertinent values [17].

### 2.4. Contact Angle (CA) Test

CA measurements were conducted utilising a tensiometer (Theta Lite, Dyne Technology, Staffordshire, UK) to evaluate alterations in CAs arising from different weight ratios of GF powder within the GIC composition (n = 8/group). The CA was ascertained via gauging the angle formed when a 3 μL water droplet was deposited on the specimen’s surface after a 20 s duration.

### 2.5. Film Thickness Test

**Figure 2:**
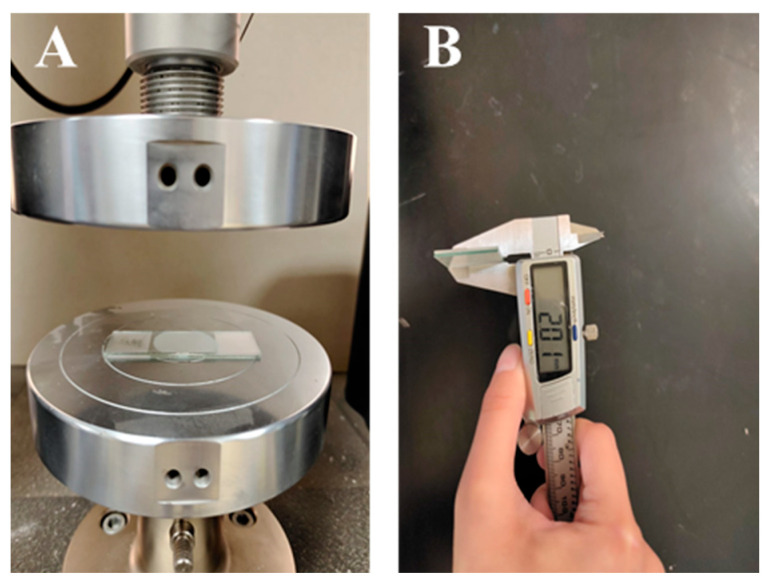
Film thickness test: (**A**) film thickness test being conducted in a universal testing machine and (**B**) varnier calliper being used to measure the film thickness.

To ascertain the film thickness, ISO standard 9917-1 was employed [18]. The control and experimental groups followed the manufacturer’s recommended dispensing and mixing protocols. Precise measurements were taken for the thickness of two glass slides, with a minimal margin of error not exceeding 0.01 μm. This measurement was established as the reference standard (A). A volume of cement mixture, precisely 0.1 ± 0.05 mL, was carefully dispensed via micropipette onto the centre of one glass slide, followed by the placement of a second glass plate on top of it, effectively enclosing the mixture. A 150 N vertical load was applied to the central region of the glass slides using a universal testing machine for 10 s. After a 10 min delay, the glass slides’ thickness was remeasured (Figure 2B). The film thickness was determined as the difference between measurements B and A. This process was repeated eight times per study group (n = 8/group) to calculate the average film thickness.

### 2.6. Nanohardness (nH) and Elastic Modulus (EM) Tests

nH and EM tests were performed with a nanoindentation technique via a nanomechanical tester (UMT1, Bruker, Santa Barbara, CA, USA). The device was equipped with a diamond indenter tip (with a radius of 100 nm). The system underwent calibration using a fused silica block, which possessed an EM of 72 Giga Pascals (GPa) to ensure accurate indenter area function and instrument compliance. Tests were conducted at 23 °C with loading and unloading rates of 2.0 mN/s and a 10 s dwell time at peak load. The maximum load applied was 20.0 mN. Both the nH and EM were computed with the help of a proprietary software integrated in the testing device. The mean values for nH and EM were determined by taking three measurements on each specimen (n = 8/group).

### 2.7. Compressive Strength (CS) Test

For CS testing, specimens from each study group (n = 8/group) were positioned with their flat ends facing upward between the plates of the universal testing machine (Instron 3369; Canton, MI, USA). A compressive force was applied to each specimen utilising a load cell of 5 kN, at a controlled crosshead speed of 0.5 mm/min, continuing until the specimen exhibited cracking. The proprietary software, namely Bluehill ver. 2.4 (Instron; Canton, MI, USA), automatically computed the CS.

### 2.8. Diametral Tensile Strength (DTS) Test

Within each experimental group (n = 8/group), the specimens were positioned so that their flat ends aligned perpendicular to the base plate of the universal testing machine (Instron 3369; Canton, MI, USA). This arrangement was employed to subject the specimens to stress along their diameter. A compressive force of 5 kN was exerted, and the compression was carried out at a controlled crosshead speed of 1.0 mm/min. The compression continued until the specimen fractured. The DTS values in mega Pascals were obtained using proprietary software (Bluehill ver. 2.4).

### 2.9. Water Sorption (Wsp) and Water Solubility (Wsol) Tests

In each group (n = 8/group), specimens were initially desiccated with silica gel for 2 h and then incubated at 37 °C for 24 h until a constant mass was reached. Their initial mass (m1) was measured using a precise electronic scale (Precisa, EP 320A; Dietikon, Switzerland) accurate to 0.1 mg. After obtaining the initial mass, the specimens were immersed in distilled water for seven days, yielding the wet mass (m2). Subsequently, they were dehydrated in an incubator at 37 °C for 24 h, and the final drying mass (m3) was measured. Wsp was calculated as (m2 − m1), and Wsol as (m1 − m3). Percentages of Wsp and Wsol were determined using the Equation:

Wsp = 100 × (m2 − m1)/m1(1)

Wsol = 100 × (m1 − m3)/m1(2)

### 2.10. Statistical Analysis

The normality of data distribution was verified through the Shapiro—Wilk test (significance level α = 0.05). Additionally, Levene’s test was conducted to assess the equality of variances. The acquired data underwent statistical scrutiny utilising SPSS version 28 (IBM Corp., New York, NY, USA). Group contrasts were executed via one-way analysis of variance (ANOVA), succeeded by Tukey’s post hoc tests, adopting a 95% confidence threshold (*p* < 0.05).

## 3. Results

### 3.1. Surface Texture 

**Figure 3:**
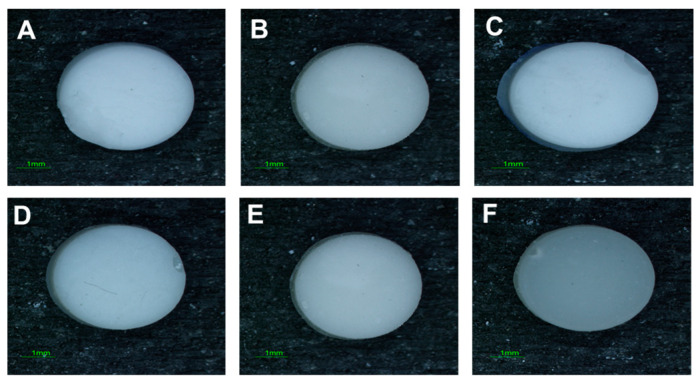
Stereomicroscope images of the study specimens: Images (**A**–**C**) depicts the control, 0.5 wt.% GF-GIC and 1.0 wt.% GF-GIC using MC, respectively. Figures (**D**–**F**) represent the control, 0.5 wt.% GF-GIC and 1.0 wt.% GF-GIC of KCR, respectively. Scale bar represents 1 mm.

The visual examination revealed no difference in the colour of test specimens. All the study specimens exhibited a consistent and uniform smooth texture. Upon visual inspection, there were no apparent irregularities, roughness, or textural variations observed (Figure 3).

### 3.2. Porosity

**Figure 4:**
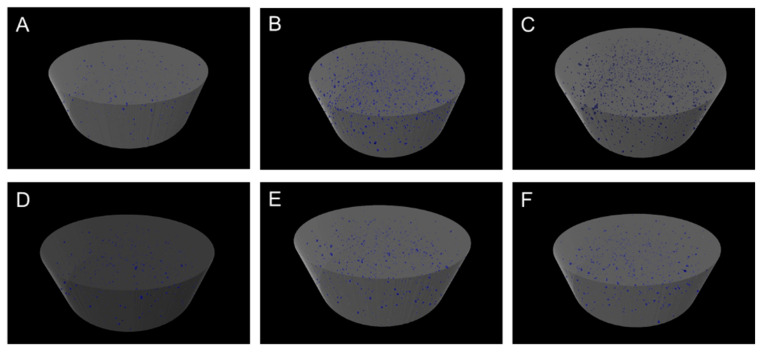
Internal porosity evaluation of experimental GIC luting materials using micro-CT. Figures (**A**–**C**) represent the control, 0.5 wt.% GF-GIC and 1.0 wt.% GF-GIC of MC, respectively. Figures (**D**–**F**) represent the control, 0.5 wt.% GF-GIC and 1.0 wt.% GF-GIC of KCR, respectively.

The control group for both materials (MC and KCR) had the lowest closed and open porosity. As the concentration of GF-reinforced GIC (GF-GIC) increased (0.5 wt.% to 1.0 wt.%), the closed porosity (pores within a material that do not have direct access to the external environment) and open porosity (pores within a material that have direct access to the external environment) generally increased. Among the two materials, MC generally exhibited higher porosity values compared to KCR, regardless of the GF-GIC concentration (Table 1, Figure 4).

### 3.3. Contact Angle (CA)

For both MC and KCR materials, the control groups (without any GF reinforcement) exhibited similar mean CAs, with values around 73–74°. When GF-GIC was added to both MC and KCR, the mean CAs increased. Among the GF-GIC groups, MC with 1.0 wt.% GF-GIC and KCR with 1.0 wt.% GF-GIC had the highest mean CAs (95.65° and 84.45°, respectively). The details are presented in Table 2.

### 3.4. Film Thickness

For both MC and KCR materials, the control groups showed different film thicknesses, i.e., MC’s control group had a film thickness of 20.5 µm, compared to KCR’s control group at 15.1 µm (Table 3). When GF-GIC was added to both materials, the film thickness increased. Among the GF-GIC groups, MC with 1.0 wt.% GF-GIC had the highest film thickness (25.6 µm), followed closely by MC with 0.5 wt.% GF-GIC (24.2 µm). Similarly, KCR with 1.0 wt.% GF-GIC had a higher film thickness (18.4 µm) than KCR with 0.5 wt.% GF-GIC (16.3 µm).

### 3.5. Nanohardness (nH) and Elastic Modulus (EM)

**Figure 5:**
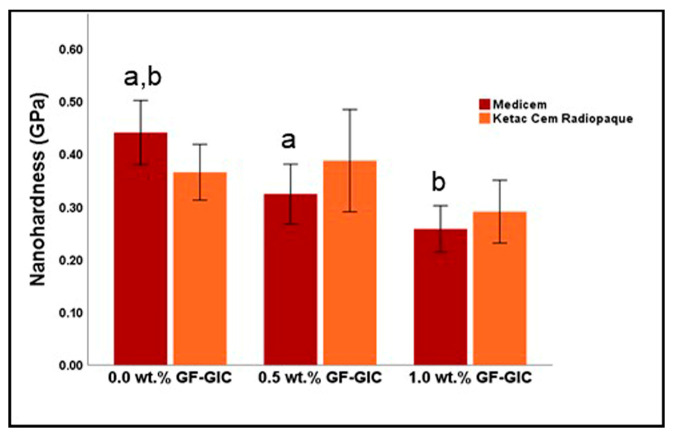
Comparison of NH (in GPa) between experimental GlC luting materials. Key: the same lower-case letters depict significant differences within the MC GIC group.

Figure 5 represents the graphical representation of the descriptive and inferential statistics related to nH (in GPa) of the study groups. The control group of MC exhibited the highest mean nH of all. The 1.0 wt.% GF-GIC group exhibited the lowest nH, indicating that adding more GF leads to reduced nH. However, it is worth noting that the 0.5 wt.% GF-GIC showed slightly higher nH compared to the control group. It also had a higher standard deviation, suggesting greater variability in nH. According to Bonferroni’s multiple comparisons tests, for both MC and KCR materials, the differences in nH between the groups were not significant, except for two cases in the MC material; the control group in MC showed significantly higher nH compared to both the 0.5 wt.% GF-GIC and 1.0 wt.% GF-GIC groups.

**Figure 6:**
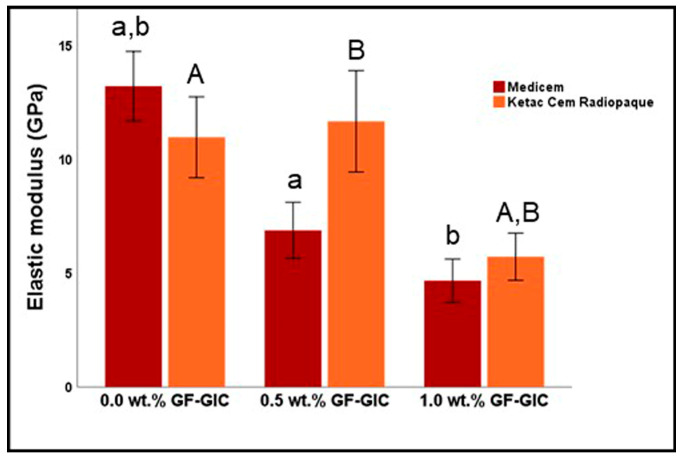
Comparison of EM (in GPa) between experimental GlC luting materials. Key: The same lower-case letters depict significant differences within the MC GIC group while upper-case letters depict significant differences with the KCR GIC group.

Figure 6 represents the graphical representation of the descriptive and inferential statistics related to EM (in GPa) of the study groups. The control group of MC exhibited the highest while 1.0 wt.% GF-GIC group exhibited the lowest EM of all. In contrast, the EM for the KCR decreased in 1.0 wt.% GF-GIC group. However, it is worth noting that the 0.5 wt.% GF-GIC showed slightly higher EM compared to the control group and had a higher standard deviation, suggesting greater variability in EM. According to Bonferroni’s multiple comparison tests, the control group showed a significant difference with 0.5 wt.% GF-GIC and 1.0 wt.% GF-GIC groups in MC GIC whereas the control and 0.5 wt.% GF-GIC groups showed significantly higher EM to 1.0 wt.% GF-GIC group in KCR.

### 3.6. Compressive Strength (CS)

**Figure 7:**
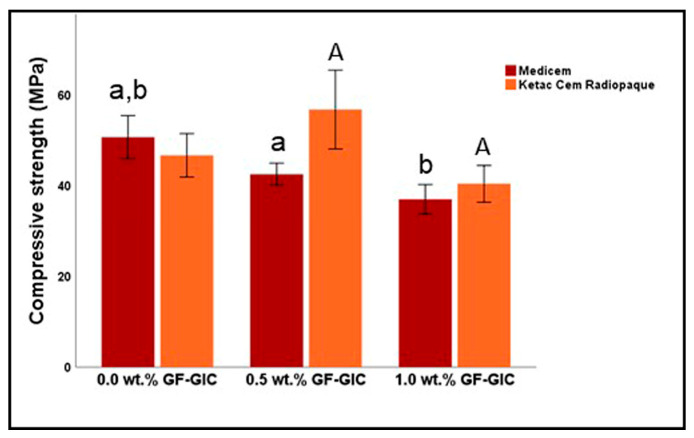
Comparison of CS (in MPa) between experimental GlC luting materials. Key: see Figure 6.

Figure 7 represents the graphical representation of the descriptive and inferential statistics related to CS (in MPa) of the study groups. The CS seemed to decrease as the wt.% of GF increased in MC GIC. The 0.5 wt.% GF-GIC of KCR exhibited the highest while 1.0 wt.% GF-GIC of MC exhibited the lowest CS of all. The CS of the KCR decreased in 1.0 wt.% GF-GIC group. However, the 0.5 wt.% GF-GIC showed higher CS compared to the control group of MC. It also had a higher standard deviation, suggesting greater variability in CS. According to Bonferroni’s multiple comparison tests, the control group showed a significant difference with 0.5 wt.% GF-GIC and 1.0 wt.% GF-GIC groups in MC GIC whereas the 0.5 wt.% GF-GIC group showed significantly higher CS to 1.0 wt.% GF-GIC group in KCR.

### 3.7. Diametral Tensile Strength (DTS)

**Figure 8:**
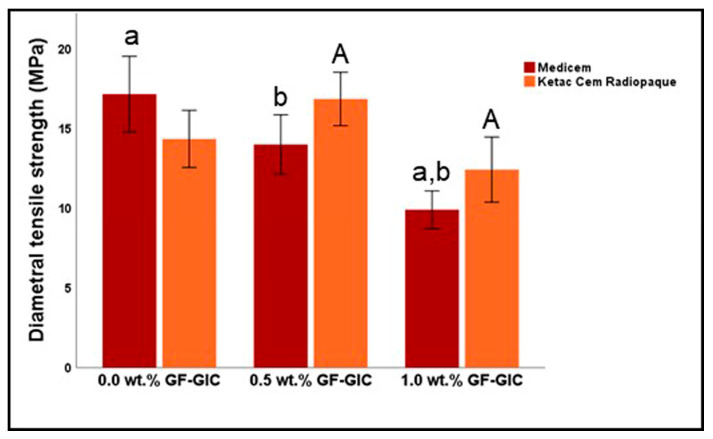
Comparison of the DTS (in MPa) between experimental GlC luting materials. Key: see Figure 6.

Figure 8 represents the graphical representation of the descriptive and inferential statistics related to DTS (in MPa) of the study groups. The DTS seemed to decrease significantly as the wt.% of GF increased in MC GIC. The control group exhibited the highest while 1.0 wt.% GF-GIC of MC exhibited the lowest DTS of all. The DTS for the KCR was observed to decrease in 1.0 wt.% GF-GIC group. However, the 0.5 wt.% GF-GIC showed higher DTS compared to the control group of MC. According to Bonferroni’s multiple comparison tests, the control and 0.5 wt.% GF-GIC groups showed significant differences with 1.0 wt.% GF-GIC in MC GIC whereas the 0.5 wt.% GF-GIC group showed significantly higher CS to 1.0 wt.% GF-GIC group in KCR.

### 3.8. Wsp and Wsol

In Table 4, both MC and KCR showed an increased tendency to solubility and *Wsp* at 1.0 wt.% GF-GIC concentration. Among the two materials, MC tended to have higher solubility and Wsp values compared to KCR. MC with 1.0 wt.% GF-GIC exhibited the highest solubility and Wsp values among all groups, indicating that higher concentrations of GF-GIC may lead to increased dissolution and water absorption. The addition of 0.5 wt.% GF to GIC of KCR reduced the Wsol and Wsp.

## 4. Discussion

The outcomes derived from this laboratory investigation partially delineate that the integration of GF into GIC manifests a potentially advantageous influence on both surface characteristics and mechanical attributes. However, the amount of GF as well as the specific GIC formulation can influence the extent of mechanical property improvement. Notably, the improvements and synergistic effect were observed to be significant using one formulation of GIC while insignificant in the other. As a result, the formulated hypothesis is hereby partially validated.

Due to a high resolution and enhanced visibility from illumination options, a stereomicroscope was used. The visual observation revealed that the surface texture remained unaffected by the presence of GF. In contrast, the microstructure evaluation using micro-CT revealed that the control groups in both materials resulted in the lowest porosity. With the increase in weight ratio of GF in GIC, there was a corresponding rise in porosity levels, signifying the possible formation of voids within the cement structure due to the presence of GFs [19]. The analysis also highlighted the influence of material composition, as MC generally exhibited a higher porosity compared to KCR. 

The water CA is commonly used as an indicator of surface hydrophobicity, with higher angles indicating greater hydrophobic behaviour [20]. The GF-GIC composites of both dental cements showed increased CAs. The effect of GF-GIC on the CA is attributed to the hydrophobic nature of GFs, which tend to repel water and create a more water-resistant surface when integrated into the cement matrix. The higher CA measurements among the experimental groups with GF suggest the hydrophobic nature of the luting material. The higher CA measurements using the GF among the experimental groups suggest improved marginal sealing and prevention of oral fluids from infiltrating the interface. The absence of previously published studies appraising the impact of GFs in GIC precludes our ability to undertake a comparative analysis and assessments of porosity and CA measurements.

The measurement of luting cement film thickness constitutes a pivotal rheological attribute facilitating the appropriate placement of restorations onto prepared teeth. According to ISO 9917–1:2007 standards, the film thickness of luting cements is mandated to not surpass 25 μm [18,21]. All the groups exhibited film thickness lower than this limit except for the composite of 1.0 wt.% GF-GIC using MC that exceeded the limit (i.e., 25.6 μm). The data relating to film thickness suggest that the powder particle size of MC GIC was larger than the powder particle size of KCR, therefore film thickness of the control group using MC GIC showed higher thickness than the control group of KCR. A progressive increase in film thickness was observed with increasing weight ratios of GF among the experimental groups of both GICs. The enhanced film thicknesses of the experimental groups compared to their respective control group suggest insolubility, resistance to spreading, and inability to blend GF in GIC [21]. The achieved results are consistent with the previous studies that advocated increased film thickness due to reinforced filler [3,21]. 

The nH and EM data is indeed interesting and provides valuable insights into the mechanical behaviour, offering a deeper understanding of how the incorporation of micron-sized GFs influences the overall strength and performance of the GIC composite. Contrary to the experimental groups of MC GIC where we observed a downward trend in nH and EM compared to the control group, we observed a synergistic effect in terms of nH and EM using 0.5 wt.% GF-GIC KCR composite. This could suggest that this particular composition of GIC provides synergetic effects with GF using 0.5 wt.%. During the acid-base reaction, a three-dimensional network or gel structure forms within the cement as a result of the creation of ionic connections between the positively charged metal ions (such as calcium and aluminium) in the powder component and the negatively charged oxygen ions in the liquid component. The GF may get enclosed in the gel matrix and improve the set cement’s mechanical characteristics. As the reaction intensifies, this structure gets harder. The GF at 0.5 wt.% might promote a more uniform distribution of particles and reduce the presence of voids or defects, leading to a more consistent and harder material. Decreased properties of 1.0 wt.% GF-GIC composites in both formulations might be attributed to a combination of factors related to fibre dispersion, interfacial bonding, matrix disruption, mechanical mismatch, and specimen variability [22,23].

The observed disparity in the enhancement of nH and EM, resulted from the incorporation of 0.5 wt.% GF in one GIC formulation as opposed to the attenuation of mechanical properties in another could be due to the base composition of the GICs that can vary significantly. Some GIC formulations might already contain additives or components that interact favourably with GF, leading to enhanced mechanical properties. On the other hand, certain compositions might have chemical interactions that interfere with the reinforcing effect of the fibres, resulting in decreased mechanical properties [3,23]. The chemical interactions at the interface between the fibres and the GIC matrix contributed to the improved mechanical properties. Our findings are in line with the previous studies that advocated the positive effect of GF on the hardness and EM of GIC [10,12].

We observed a consistent correlation in the alterations of properties across various groups, encompassing both compression and DTS, similar to what was noted in the changes observed in nH and EM. The rationale for the reduction in CS and DTS resulted from the inclusion of 1.0 wt.% GF in GIC was elucidated earlier, as was the rationale for the augmented CS and DTS stemming from the incorporation of 0.5 wt.% GF in one GIC formulation while experiencing synergistic effects in another. However, a higher aspect ratio of GF, i.e., 11:1 was used for the enhanced mechanical properties and we observed improvement in some formulations. In principle, optimal outcomes also hinge upon aligning fibres parallel to the primary load direction [15]. Nonetheless, real-world circumstances pose a challenge due to the inclination of short fibres to disperse in a stochastic manner, adopting diverse orientations throughout the mixing process. Consequently, the gains in material strength arising from this random fibre dispersion typically manifest as marginal improvements [22]. However, according to ISO standard (ISO 9917-1), more than 50 MPa and 15 MPa are required for CS and DTS, respectively [18]. We observed that the control group of MC and 0.5 wt.% GF-GIC group of KCR achieved this target.

The longevity of cement was significantly influenced by essential factors, namely Wsp and Wsol, which are closely interlinked [11,24]. In both GICs, the presence of GF appears to result in heightened Wsol and Wsp, as evident in the 1.0 wt.% GF-GIC groups. Conversely, in certain instances, such as the 0.5 wt.% GF-GIC groups of the KCR material, the Wsol and Wsp appear to be reduced compared to the control. The reason could be the synergistic effect and improved cohesion between GF and glass particles of GIC. The presence of GF might densify the material’s microstructure, decreasing the void spaces that can trap water. Due to the hydrophobic nature and small surface area of GF in comparison to finer GIC particles, a reduced number of sites available for water interaction, ultimately results in a decline in Wsol and Wsp. Additionally, GF possesses a hydrophobic nature. This hydrophobicity could limit water penetration into the material, resulting in reduced Wsp and solubility. However, at 1.0 wt.% GF, the matrix of GIC may get disrupted and uniform dispersion of GF becomes challenging. Therefore, the properties are compromised. 

These findings contribute to a better understanding of the microstructural characteristics of GF-GIC composites and aid in optimizing their properties for various practical applications. Future research can further investigate the relationship between porosity and mechanical performance to provide a comprehensive understanding of the material’s behaviour and facilitate its optimal utilisation. Laboratory studies often focus on short-term observations, which might not capture the full extent of material behaviour over longer periods. Effects such as degradation, wear, and ageing might not manifest within the study’s duration. A combination of laboratory studies, computational modelling, and clinical trials is often necessary for future research.

## 5. Conclusions

In the context of the constraints inherent to this laboratory investigation, we can conclude that the inclusion of silanized GF could potentially yield a synergistic effect on the mechanical attributes of KCR GIC using 0.5 wt.% GF. Silanized GF can be used as a functional additive in GIC luting material with promising results. However, due to variations in the composition of GIC, it is possible that silanized GF may not exert an effective reinforcing influence in all the formulations, as observed in KCR GIC.

## Figures and Tables

**Table 1:** Porosity analysis of dental luting cements with GF-reinforced GIC additives.

Material	Group	Closed Porosity (%)	Open Porosity (%)	Total Porosity (%)
MC	Control	0.10	22.17	22.27
	0.5 wt.% GF-GIC	1.52	28.59	30.11
	1.0 wt.% GF-GIC	3.61	29.61	33.22
KCR	Control	0.18	24.56	24.74
	0.5 wt.% GF-GIC	0.58	25.13	25.71
	1.0 wt.% GF-GIC	0.41	27.05	27.46

**Table 2:** Mean CA (°) of various dental luting cements with GF-reinforced GIC Additives.

Group	Mean CA (°)	Image
MC (control)	73.37 ± 3.63	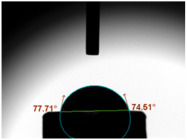
MC 0.5 wt.% GF-GIC	92.90 ± 1.58	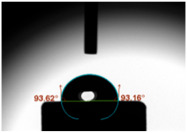
MC 1.0 wt.% GF-GIC	95.65 ± 2.19	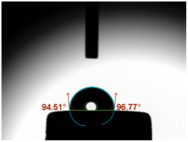
KCR (control)	73.72 ± 1.53	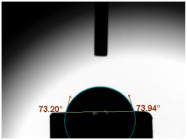
KCR 0.5 wt.% GF-GIC	77.12 ± 4.37	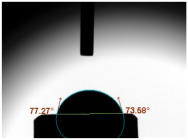
KCR 1.0 wt.% GF-GIC	84.45 ± 1.37	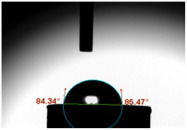

**Table 3:** Film thickness (µm) of dental luting cements with GF-reinforced GIC additives.

Group	Film Thickness (µm)	
	MC	KCR
Control	20.5 ± 3.1	15.1 ± 3.0
0.5 wt.% GF-GIC	24.2 ± 3.4	16.3 ± 3.3
1.0 wt.% GF-GIC	25.6 ± 4.6	18.4 ± 2.9

**Table 4:** Wsol and Wsp (%) of dental luting cements with GF-reinforced GIC additives.

Group	MC		KCR	
	Wsol (%)	Wsp (%)	Wsol (%)	Wsp (%)
Control	0.27 ± 0.22 ^a^	6.62 ± 0.94 ^a^	0.22 ± 0.10 ^a^	2.63 ± 0.48
0.5 wt.% GF-GIC	0.61 ± 0.33 ^a^	8.71 ± 0.82 ^a,b^	0.19 ± 0.08 ^a^	1.68 ± 0.41 ^a^
1.0 wt.% GF-GIC	1.75 ± 0.86 ^b^	9.25 ± 1.21 ^b,c^	0.46 ± 0.22 ^b^	2.83 ± 0.51 ^a,b^

Key: The same lower-case letters depict significant differences within the column groups.

## Data Availability

The data presented in this study are available on request from the corresponding author.
